# Editorial: Greening urban spaces and human health, volume II

**DOI:** 10.3389/fpubh.2025.1594929

**Published:** 2025-06-05

**Authors:** Abdullah Akpınar, Yuan Li, Ding Li

**Affiliations:** ^1^Department of Landscape Architecture, Faculty of Agriculture, Aydin Adnan Menderes University, Aydın, Türkiye; ^2^School of Geography and Tourism, Shaanxi Normal University, Xi'an, Shaanxi, China; ^3^Northwest Land and Resources Research Center, Shaanxi Normal University, Xi'an, Shaanxi, China; ^4^School of Public Administration, Southwestern University of Finance and Economics, Chengdu, China

**Keywords:** urban green spaces, human health, psychological well-being, urban planning, nature-based solutions

## Introduction

Urbanization continues to transform the global landscape, with more than half of the world's population now living in cities and that proportion expected to rise to nearly 70% by 2050 (1). While urban living offers many benefits—economic opportunity, cultural vibrancy, and innovation—it also brings significant public health challenges, such as exposure to pollution, rising temperatures, and reduced access to natural environments (2). In this context, urban green spaces have been increasingly recognized for their potential to contribute to healthier, more sustainable cities (3, 4). However, while evidence on their benefits continues to grow, critical questions remain about how, when, and for whom these benefits are realized.

This second volume of the Research Topic *Greening Urban Spaces and Human Health* brings together 28 studies that extend our understanding of the relationship between urban nature and health. The contributions span diverse geographies, disciplines, and methods. Rather than summarize each individual paper, we take this opportunity to highlight a few key themes and persistent challenges that emerge from this body of work.

## Urban green spaces as health interventions: promise and complexity

Several studies in this Research Topic support the health-promoting potential of green spaces. Evidence links green infrastructure to reductions in stress, improved mental health, and increased physical activity. However, translating these benefits into effective health interventions is not straightforward. Benefits vary depending on the quality, accessibility, and perception of green spaces, as well as on individual and contextual factors. As such, green space interventions must be carefully designed, tailored, and evaluated within specific urban contexts. The idea that green spaces “may” serve as essential infrastructure for health—depending on multiple mediating factors—must guide future research and policy.

## Measuring what matters: methodological gaps and opportunities

This volume also highlights ongoing challenges in defining and measuring both green space and health outcomes. Some reviews and empirical studies point to inconsistencies in how greenery is quantified (e.g., street-level greenery, park proximity, and biodiversity) and how health is assessed (e.g., self-reports, physiological markers, and hospital admissions). These inconsistencies complicate comparisons across studies and limit the generalizability of findings. Future research would benefit from greater methodological rigor, transparency, and the use of standardized metrics, as well as mixed-method approaches that account for subjective experiences and community preferences.

## Equity and justice in urban greening

A recurring issue across multiple papers is the uneven distribution of urban greenery and the resulting disparities in health benefits. Lower-income and marginalized communities often have less access to high-quality green spaces, reinforcing environmental injustices. Some articles explore concepts like Green Space Justice and advocate for planning strategies that prioritize equity in both green space provision and design. These insights underline the importance of aligning urban greening initiatives with social justice goals and inclusive community engagement.

## Integrating nature into urban systems: interdisciplinary and policy implications

The collected studies point to the importance of interdisciplinary thinking in urban sustainability. Contributions address not only the direct effects of greenery on health, but also its roles in economic resilience, mobility, public communication, and climate adaptation. Several papers examine how urban green infrastructure intersects with broader policy frameworks, such as environmental taxation, industrial innovation, or crisis response strategies. This reinforces the notion that greening efforts must be embedded within wider urban systems thinking and governance.

Taken together, the contributions in this volume deepen our understanding of the diverse ways urban nature intersects with human wellbeing. They also point to critical areas for future work: refining measurement tools, addressing equity, contextualizing interventions, and fostering interdisciplinary collaboration. Urban green spaces may offer a powerful means of promoting health and sustainability—but only when their planning, design, and implementation are informed by nuanced, evidence-based, and context-sensitive approaches.

We thank all contributors for their important work and encourage readers to explore the individual articles in this Research Topic. We hope that the insights offered here stimulate further dialogue, research, and action toward greener, healthier, and more equitable urban futures.
